# Ubiquitous News Coverage and Its Varied Effects in Communicating Protective Behaviors to American Adults in Infectious Disease Outbreaks: Time-Series and Longitudinal Panel Study

**DOI:** 10.2196/64307

**Published:** 2025-03-10

**Authors:** Anqi Shao, Kaiping Chen, Branden Johnson, Shaila Miranda, Qidi Xing

**Affiliations:** 1 Department of Life Sciences Communication, University of Wisconsin - Madison Madison, WI United States; 2 Decision Research Eugene, OR United States; 3 Department of Information Systems, University of Arkansas Fayetteville, AR United States; 4 School of Computing and Analytics, Northern Kentucky University Highland Heights, KY United States

**Keywords:** risk communication, panel study, computational method, intermedia agenda setting, protective behaviors, infectious disease

## Abstract

**Background:**

Effective communication is essential for promoting preventive behaviors during infectious disease outbreaks like COVID-19. While consistent news can better inform the public about these health behaviors, the public may not adopt them.

**Objective:**

This study aims to explore the role of different media platforms in shaping public discourse on preventive measures to infectious diseases such as quarantine and vaccination, and how media exposure influences individuals’ intentions to adopt these behaviors in the United States.

**Methods:**

This study uses data from 3 selected top national newspapers in the United States, Twitter discussions, and a US nationwide longitudinal panel survey from February 2020 to April 2021. We used the Intermedia Agenda-Setting Theory and the Protective Action Decision Model to develop the theoretical framework.

**Results:**

We found a 2-way agenda flow between selected national newspapers and the social media platform Twitter, particularly in controversial topics like vaccination (*F*_1,426_=16.39; *P*<.001 for newspapers; *F*_1,426_=44.46; *P*<.001 for Twitter). Exposure to media coverage increased individuals’ perceived benefits of certain behaviors like vaccination but did not necessarily translate into behavioral adoption. For example, while individuals’ media exposure increased perceived benefits of mask-wearing (β=.057; *P*<.001 for household benefits; β=.049; *P*<.001 for community benefits), it was not consistently linked to higher intentions to wear masks (β=–.026; *P*=.04).

**Conclusions:**

This study integrates media flow across platforms with US national panel survey data, offering a comprehensive view of communication dynamics during the early stage of an infectious disease outbreak. The findings caution against a one-size-fits-all approach in communicating different preventive behaviors, especially where individual and community benefits may not always align.

## Introduction

### Background

The shift from traditional to digital media marks a significant change in how the public accesses scientific information, with social media emerging as a central platform [1]. Media coverage may amplify or attenuate risk judgments of its audiences [2], increase risk perception of disease outbreaks [3], and encourage them to take protective actions [4]. Although previous research has explored the relationship between media exposure, risk perception, and protective behaviors during infectious disease outbreaks, the specific impact of distinct media content on the public’s adoption of protective behaviors remains unclear. This study aims to bridge this gap by examining how exposure to specific media contents from different sources might alter people’s perspectives and willingness to adopt protective measures, building upon but distinct from the broader media agenda-setting literature.

### The Role of Different Media in Communicating Preventative Behavior Through the Lens of Intermedia Agenda-Setting Theory

During infectious disease outbreaks, nationwide health messages are warranted for successfully building up healthier collective behavior. It is assumed that such messages, including those to overcome the COVID-19 pandemic, would work if they are clear [5], consistent [6], and come from reliable, nonpolitical sources [7]. These messages typically include descriptions of and recommendations to enact, various protective behaviors for COVID-19. Yet the term “protective behavior” during the pandemic was often used too broadly, covering both simple actions like handwashing and more complex ones like getting vaccinated. It is difficult to effectively address the questions and concerns raised by such varied actions in single messages, or even a single information campaign. This problem is further complicated by other challenges of public health communication, such as engaging diverse communities [8], stigmatization [9], and misinformation, such as antivaccination propaganda [10,11].

Since early 2020, the US Centers for Disease Control and Prevention (CDC) issued guidelines for protective behaviors against COVID-19, such as handwashing and mask-wearing [12]. While some behaviors like mask mandates were communicated top-down from authorities to the public [13], others, like vaccination debates before the widespread availability of COVID-19 vaccines in December 2020, first emerged on social media and then flowed upward (ie, bottom-up) to legacy media [14]. As communication about protective behaviors happens across different media types, it is crucial for scholars to understand the transition of information between traditional media outlets, such as newspapers and television, and social media platforms like Facebook and X (formerly Twitter, during our analysis period).

However, it remains unclear whether these social media discussions are driven by traditional media outlets such as newspapers’ coverage or vice versa. Understanding this relationship is crucial because behaviors like mask-wearing and quarantine mandates have different properties that affect their perceived benefits and costs, and may therefore have different origins and dissemination pathways for information about them. Gaining such understanding is key to comprehending how media shapes public health behavior. This leads to our first research focus: investigating the reciprocal influence between legacy newspapers and social media in setting the agenda for COVID-19 protective behaviors. This study specifically looks at the representation of these behaviors, from basic handwashing to complex vaccination debates, in selected national newspapers and on Twitter to discern the pattern and direction of this information flow. The first research question thus aims to dissect this interplay between selected national newspapers, as examples of legacy media, and Twitter, as an example of social media, in setting the agenda for infectious disease protective behaviors, RQ1: in what direction did the agenda for COVID-19 protective behaviors move between the selected top US national newspapers and Twitter?

Intermedia agenda setting serves as a framework for understanding how various news sources influence one another and the consequent breadth of information presented to the public [15]. Originating in political communication, this theory has been applied to health topics to examine the dynamics between traditional and emerging media sources. It highlights the “co-orientation” of narratives within media, influenced by both economic and local social factors [16]. This interaction affects how issues, including health, are framed and perceived, impacting public opinion and decisions. The theory explains how news content transfers between media, illustrating how dominant sources can shape the agendas of others, thereby influencing the entire media landscape [16,17]. For instance, in health communication, significant agenda flows from social media to traditional media have been observed for topics like the Measles, Mumps, and Rubella vaccine-autism debate [18] and climate change [19].

### From Communication to Action: How Media Exposure Might Influence People’s Protective Behavior

While media agendas on infectious diseases may influence public risk perceptions [20], the evidence of their impact on protective behaviors during pandemics is mixed, particularly in terms of media source and effect. First, there is limited recent evidence on how legacy media use impacts behavioral adaptations. Increased time spent on legacy media can lead to heightened anxiety and stress, which may result in misplaced health-protective behaviors [21]. Official government media use tends to encourage protective actions, while commercial media often results in overprotective behaviors driven by anxiety [22]. Notably, legacy media coverage, including national and local newspapers, television, and radio, has a significant influence on the protective actions of less-educated individuals [23]. Additionally, past research has shown that merely relying on massive media coverage of infectious diseases is not sufficient for promoting protective actions, as other factors like social norms and perceived threats also play crucial roles [24-26].

Second, although social media are key tools for disseminating health messages and can positively influence health-related behaviors [27-31], their effectiveness in behavior change is not consistent. For instance, increased exposure to media content about Ebola heightened risk perceptions, but a similar increase in exposure to social media content about the Zika virus did not shift risk perceptions among mainland Americans [3,20]. Furthermore, evidence suggests a negative relationship between health-protective behaviors and social media use, where heightened concerns and a lower construal level associated with social media do not encourage preventive actions but instead reinforce negative emotional reactions and mental health problems [32].

In this research, we drew from the Protective Action Decision Model (PADM) for a comprehensive theoretical framework to understand how media agendas of protective behaviors relate to actual individual practices [33]. The PADM framework details how decision-making begins with environmental cues like sight and smell, social cues observed from the behavior of others, and warnings from information sources [33,34]. It specifies prompts for action that are crucial for encouraging engagement in health-related behaviors during infectious disease outbreaks such as H7N9 [35]. The PADM has been applied to the COVID-19 context with a focus on the early stages [36], and in different cultural backgrounds [37,38].

This study collected panel survey data from around 2000 American adults to examine the relationship between media exposure and individuals’ behavioral intentions to protect themselves during the COVID-19 pandemic. The dataset spans from early in the US COVID-19 pandemic experience (February 2020) to the period after vaccines became widely available (April 2021). In line with the PADM, which considers intentions as a function of risk perceptions, behavior perceptions, and stakeholder perceptions, this study focuses specifically on behavior perceptions. Since our media content analysis primarily dealt with protective behaviors rather than the overall COVID-19 threat or trust in institutions, we chose to emphasize how respondents perceive the benefits of these behaviors. Based on PADM, we developed our main hypothesis as H1: US individuals’ perceived benefit of protective behaviors is positively associated with their media exposure to those behaviors.

We argue that media exposure’s impact on behavior is not straightforward but mediated through perceived benefits. According to the original model by Lindell and Perry, before behavior adoption, individuals assess it based on media, evaluating factors such as effectiveness, safety, and relevance. Their perception of these benefits is a crucial factor in deciding whether to adopt certain behaviors, while perceived barriers (such as resource limitations) can hinder the translation of intention into action. During the COVID-19 pandemic, media exposure indirectly influenced health behaviors by promoting protective actions in public spaces through combined perceptions of social initiatives and moderate fear [39], and by positively affecting perceived efficacy and threat awareness [40], leading to improved health-protective behaviors. In other contexts, exposure to health-relevant messages indirectly influences behavioral intentions, such as perceived social norms affecting binge drinking [41] or perceived benefits encouraging regular exercise [42]. This body of evidence, illustrating how media exposure may shape behavior through the lens of perceived benefits, lays the foundation for our second hypothesis H2: the association between media exposure to protective behaviors and the actual practice of these behaviors is mediated by individuals’ perceived benefits of these behaviors.

## Methods

This section describes our data collection, measurements, and analysis methods for testing our hypotheses.

### Data Collection

#### Example Legacy Media and Social Media Contents

In this study, we used major national newspapers as examples for legacy media, and Twitter as an example for social media. We selected three major US newspapers: the New York Times (NYT), Wall Street Journal (WSJ), and USA Today, due to their high nationwide circulation numbers [43]. This collection of newspapers also represents a range of political ideologies—USA Today is considered centrist, The WSJ leans right of center, and The NYT leans left of center [44]. We collected newspaper coverage of the COVID-19 pandemic via the news data archive Factiva database by searching for the keywords “coronavirus” or “covid-19” between January 2020 and June 2021. Social media data were collected from Twitter using daily searches for the same keywords and within the same time period. While we aimed to focus on US-based conversations, our Twitter dataset may include tweets from English-speaking users outside the United States, as geo-tagging is not always available. In total, we collected 41,235 news articles and 2,398,046 tweets in English mentioning either or both of the keywords “coronavirus” or “covid-19.”

**Figure 1:**
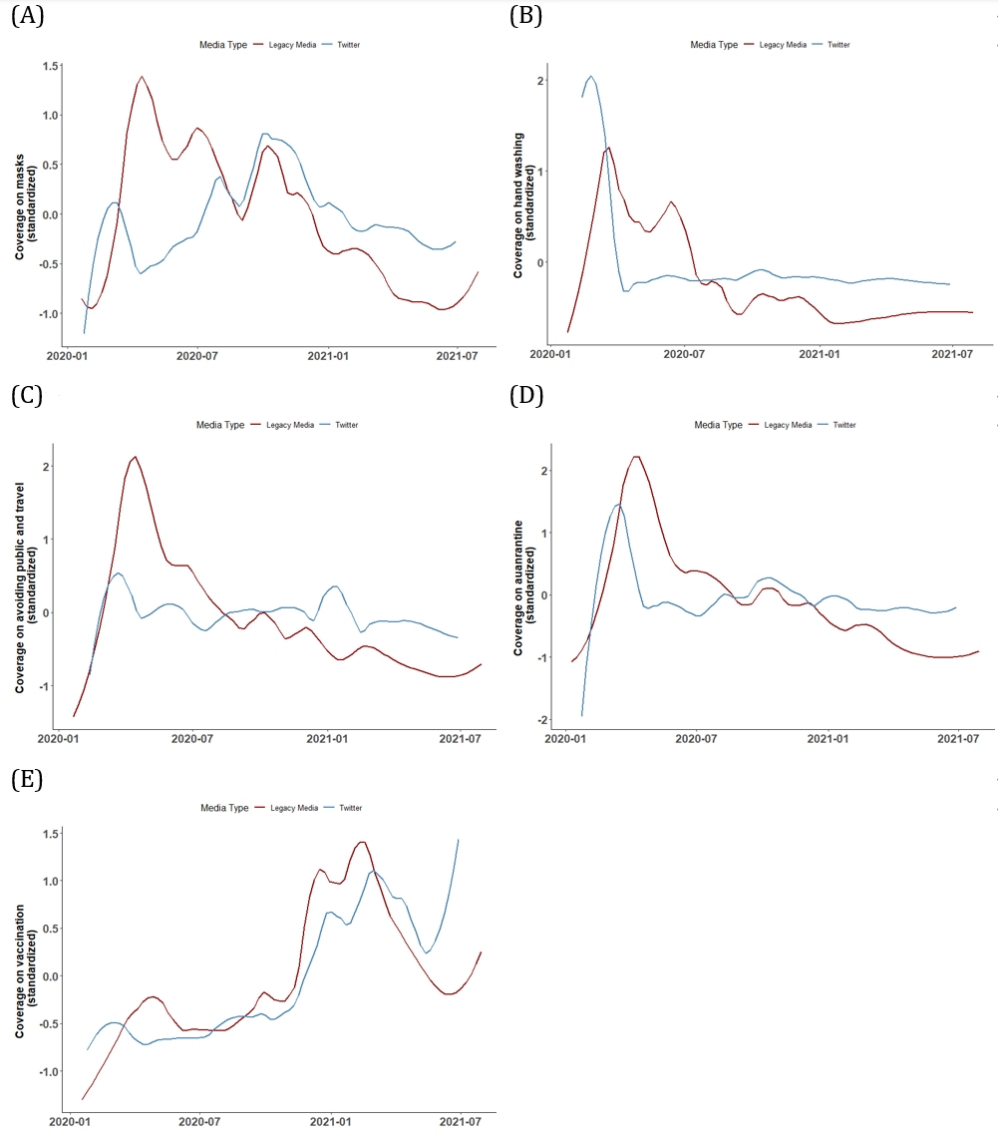
Standardized coverage from newspapers and Twitter on protective behaviors (the raw volume of daily Twitter coverage is much greater than that from newspapers. Therefore, we conducted a scaling process [ie, calculating z scores] for each set of coverage to make the figure clearer. We also smooth the trendlines with the spline function for better aesthetic quality) over time including (A) mask-wearing, (B) handwashing, (C) avoiding large public gatherings and traveling, (D) isolation at home (self-quarantine), and (E) getting vaccinated. The red lines represent the volumes of legacy media content (specifically from selected national newspapers), and the blue dashed lines represent the volumes of Twitter content.

We cleaned the media content data by filtering out those irrelevant to protective behaviors. For initial filtering, we used a regular expression search based on a lexicon that covers all protective behaviors suggested by the CDC, such as handwashing and mask-wearing (Multimedia Appendix 1). However, we recognized that keyword-based filtering alone might miss the broader themes in the media content. Therefore, we used structural topic modeling using the “stim” package in R (R Project for Statistical Computing), with the publication date serving as a covariate [45]. This additional step helped us identify 47 topics in news articles and 20 in tweets (the numbers of topics were decided by the searchK diagnosis function in the R “stm” package). Based on several criteria (Multimedia Appendix 2), we selected models of size 47 topics for newspapers and 20 for Twitter posts as optimal topic models). We then excluded content that was not closely related to health, such as international politics or entertainment (for the human validation process, see Multimedia Appendix 2). In the end, 10,528 news articles and 209,261 tweets met our criteria for health-focused protective behaviors. Figure 1 visually represents how these data trends evolved over time.

#### Public Opinion Panel Survey

We recruited 2005 American adults from the Prolific digital survey panel for a longitudinal study spanning 1 year and 2 months, consisting of six waves. In this longitudinal design, respondents from each completed wave were invited to the next wave, with the final wave open to all initial respondents. Each wave lasted about 2 weeks, except for the first. Data collection occurred on February 28-29, 2020 (wave 1), April 27-May 6, 2020 (wave 2), August 5-13, 2020 (wave 3), October 12-22, 2020 (wave 4), January 22-February 11, 2021 (wave 5), and March 25-April 13, 2021 (wave 6). However, not all respondents completed every wave or answered all questions, resulting in some variation in response rates across different waves. We had 2002 responses in wave 1, a total of 1613 responses in wave 2, a total of 1197 responses in wave 3, a total of 1026 responses in wave 4, a total of 866 responses in wave 5, and a total of 1025 responses in wave 6.

Respondents were asked for their intentions about each protective behavior on a 6-point Likert scale from “My household has never considered taking this action” to “My household has taken this action and will continue to take this action as needed.” To assess respondents’ media exposure to major national newspapers such as the NYT, WSJ, and USA Today, and for the social media platform Twitter, we asked them about the frequency of using these media sources for COVID-19–related information. Some examples of survey questions can be found in Multimedia Appendix 3.

**Figure 2:**
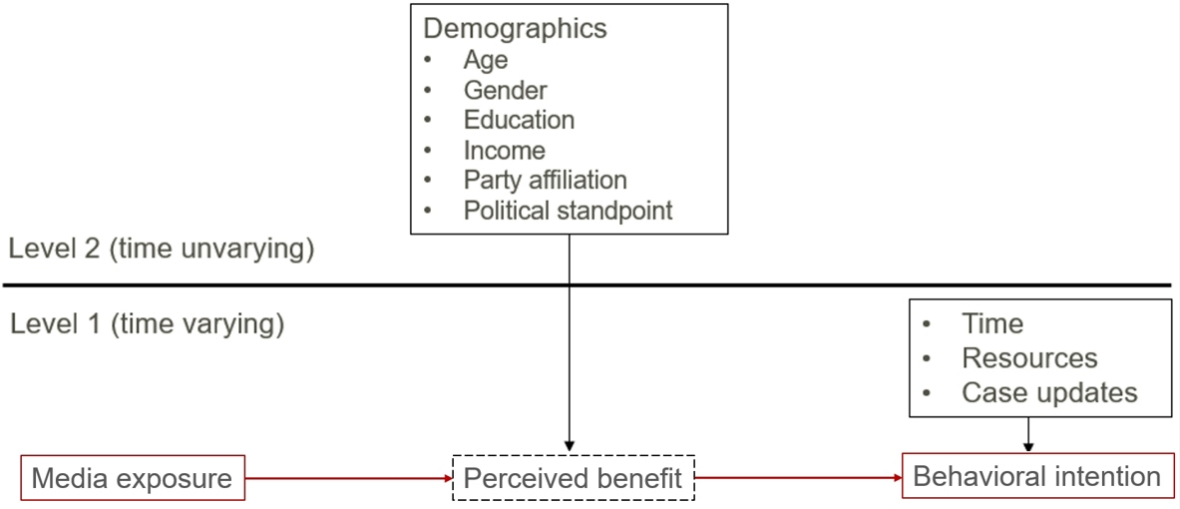
Multilevel regression model visualization, with independent variables categorized into time-varying (eg, media exposure, perceived benefits, case updates) and time-unvarying factors (eg, demographics), with fixed and random effects to capture perceptual and behavioral changes over time.

To measure respondents’ perceived benefit of protective behaviors, we asked how beneficial they think it is to practice each protective behavior in reducing risks for their household, and separately for vulnerable people in their community. To assess potential barriers to behavioral intentions, we asked respondents if they had sufficient resources to practice protective behaviors like mask-wearing and handwashing. Perceived barriers were coded as “resources” and were included in our analysis as a control on intentions, as shown in Figure 2. We also recorded demographic information, including age and gender, during the first wave.

### Ethical Considerations

Data collection from human participants was approved as exempt by the Decision Research institutional review board chair. After reading an informed consent form at each wave, outlining survey aims, benefits, risks, and other relevant information, consent was indicated by the respondent checking a box for consent. Anyone who checked the “No” box was not allowed to proceed to the survey itself. The prolific panel operator recruited all participants at wave 1, using a compensation scheme that is proprietary (ie, researchers were not informed of its type or amount), and reported all responses to researchers with a unique identifier, so that researchers were unaware of the identity of any respondent. While the survey was not password-protected, it was a closed survey in practice, as only responses from participants with matching unique identifiers were included in this analysis.

### Granger Predictive Relationship in Time Series (RQ1)

The Granger causality test is a statistical tool commonly used to infer whether one time series can predict another. It does not establish true causality but rather identifies predictive relationships between variables. This method is often used in intermedia agenda-setting research to examine the lagged predictive influence of one platform’s agenda on another [17,46,47].

The Granger causality test requires data to be stationary, as it would otherwise produce unreliable results [48]. Before running this test, we used the augmented Dickey-Fuller stationarity check, which sets a null hypothesis that the data series is nonstationary. Our tests yielded *P*-values less than .05, leading us to reject the null hypothesis for each data series. Therefore, we used Granger tests to explore the predictive relationship between daily newspaper coverage and Twitter activity for each protective behavior. We used daily coverage summary data for both newspapers and Twitter and conducted Granger tests in both directions, that is, from newspaper coverage to Twitter activity and vice versa. This allowed us to examine the predictive influence of each platform’s agenda on the other for various protective behaviors.

In this study, statistical analyses were run on R (version 4.1.2), and the time-series analyses in this session were conducted with open-sourced packages “tseries” [49] for the stationarity check and “lmtest” for Granger causality detection [50].

### Modeling Respondents’ Media Exposure and Protective Behavior (H1)

Comprehensive media coverage of protective behaviors does not indicate everyone gets the same information. To avoid making arbitrary assumptions about individual media exposure, we chose not to solely rely on either “media coverage” or survey-reported “media exposure frequency.” Instead, we calculated a “weekly media exposure” metric for each respondent by multiplying their self-reported frequency of using specific media outlets, like the NYT or Twitter, with the daily proportion of relevant coverage from those outlets. To estimate this weekly media exposure, we used one-week “exposure windows” prior to each survey wave. For each type of protective behavior, such as mask-wearing or handwashing, we calculated the specific daily proportion of coverage related to that behavior in selected top national newspapers and Twitter. Therefore, the media exposure metrics for each respondent are dynamic across different waves of survey and different types of protective behaviors. Details for calculating the media exposure can be found in Multimedia Appendix 4.

According to the PADM, the behavioral response may also be impacted by situational facilitators [33]. For situational facilitators in this study, we referred to the Center for Systems Science and Engineering at Johns Hopkins University for daily confirmed case updates in the United States from January 2020 to June 2021 [51]. More specifically, we chose to present average weekly and monthly trends of their local case updates (ie, the gradient of daily confirmed cases in the same zip code area) before each response date.

We constructed our model with multilevel modeling. As shown in Figure 2, we categorized the independent variables into two sets. The first set includes time-varying variables like respondents’ media exposure, perceived benefits, and situational factors, which change with each survey wave. The second set includes time-unvarying variables like demographics, which remain constant throughout the survey. In total, we created five models, each corresponding to one of the five protective behaviors previously mentioned. To test hypothesis H1, we examined the relationship between media exposure and perceived benefits for each protective behavior. For H2, we also looked at how these perceived benefits influence the intention to practice these behaviors. We chose the “lme4” package [52] in R because it can handle our multilevel data, which includes fixed effects like demographics and random effects showing changes in respondents across surveys. This package is effective for modeling individual changes in behavior and perception over time, thus providing a more unbiased view of how behavior changes due to media exposure and perceived benefits. To ensure comparability and interpretability of coefficients, numeric variables were standardized before analysis.

## Results

### Intermedia Agenda Setting Between Selected National Newspapers and Twitter

Table 1 presents the results of how newspaper coverage of various protective behaviors might predictably influence Twitter discourse. Table 2 shows the reverse results about how Twitter activity might predictably influence newspaper coverage.

**Table 1:** Granger test results (F statistics) of predictive causality from newspapers to Twitter posts about protective behavior issue agendas.

Lag (in days)	Wash hands, *F* test (df)	Wear masks, *F* test (df)	Self-quarantine, *F* test (df)	Avoid gathering and travel, *F* test (df)	Take vaccination, *F* test (df)
1	3.04 (1, 163)	0.19 (1, 439)	3.62 (1, 434)	1.51 (1, 368)	16.39 (1, 426)^a^
2	0.52 (2, 131)	0.18 (2, 416)	1.19 (2, 410)	0.28 (2, 343)	14.54 (2, 398)^a^
3	0.45 (3, 110)	0.28 (3, 402)	0.63 (3, 398)	0.19 (3, 331)	8.17 (3, 381)^a^
4	0.85 (4, 92)	1.04 (4, 392)	0.44 (4, 392)	0.25 (4, 321)	4.97 (4, 371)^a^
5	0.69 (5, 77)	0.86 (5, 390)	0.57 (5, 386)	0.26 (5, 312)	3.89 (5, 361)^a^

^a^*P*<.001.

**Table 2:** Granger test results (F statistics) of predictive causality from Twitter posts to newspapers about protective behavior issue agendas.

Lag (in days)	Wash hands, *F* test (df)	Wear masks, *F* test (df)	Self-quarantine, *F* test (df)	Avoid gathering and travel, *F* test (df)	Take vaccination, *F* test (df)
1	2.75 (1, 163)	0.14 (1, 439)	9.68 (1, 434)^a^	1.57 (1, 368)	44.46 (1, 426)^b^
2	0.99 (2, 131)	0.34 (2, 416)	4.62 (2, 410)^c^	1.54 (2, 343)	19.05 (2, 398)^b^
3	0.57 (3, 110)	0.37 (3, 402)	3.14 (3, 398)^c^	0.42 (3, 331)	11.81 (3, 381)^b^
4	2.14 (4, 92)	0.48 (4, 392)	2.72 (4, 392)^c^	0.41 (4, 321)	9.31 (4, 371)^b^
5	1.45 (5, 77)	0.41 (5, 390)	1.46 (5, 386)	0.70 (5, 312)	7.58 (5, 361)^b^

^a^*P*<.01.

^b^*P*<.001.

^c^*P*<.05.

Our first research question (RQ1) explored the relationship between selected national newspapers and Twitter in shaping agendas around protective behaviors. Contrary to our expectations, the results do not support a clear predictive relationship in either direction for certain behaviors like handwashing (*F*_1,163_=3.04; *P*=.08), mask-wearing (*F*_1,439_=0.19; *P*=.66), and avoiding gatherings (*F*_1,368_=1.51; *P*=.22). In these cases, no significant patterns were observed, suggesting a lack of agenda influence between newspapers and Twitter.

However, for self-quarantine, Twitter activity was found to predictably influence subsequent newspaper coverage (*F*_1,434_=9.68; *P*=.002 at a one-day lag). In the case of the more contentious, high-impact behavior of vaccination, a reciprocal relationship was observed: newspaper coverage appeared to shape Twitter discussions (*F*_1,426_=16.39; *P*<.001 at a one-day lag), and Twitter activity, in turn, seemed to influence newspaper coverage (*F*_1,426_=44.46; *P*<.001 at a one-day lag).

### Predicting Behavioral Response With Media Coverage and Real-World Data

To address the variability in behavioral responses to media coverage of protective behaviors, this section presents a detailed examination of two hypotheses. Our first hypothesis (H1) proposed that individuals’ perceived benefit of protective behaviors increases with their media exposure to these behaviors. Additionally, in our second hypothesis (H2) based on PADM, we expected that people’s perceived benefit of protective behaviors is positively related to their media exposure. Here, we observed varied patterns from our multilevel regression in the relationship between respondents’ media exposure, perceived household benefits, perceived community benefits, and behavioral intention (Bintn; Multimedia Appendix 5).

Access to resources (β=.097; *F*_1,5812_=64.40; *P*<.001) and perceived household benefits (β=.294; *F*_1,5812_=330.55; *P*<.001) significantly predicted handwashing behavior. Contrarily, perceived community benefits did not significantly influence handwashing (β=–.010; *F*_1,5812_=0.50; *P*=.48). Therefore, H1 is only partially supported in the context of handwashing behavior. Besides, the weekly trend of increasing disease cases also positively predicted handwashing (β=.024; *F*_1,5812_=5.69; *P*=.02), reflecting behavior change in response to case numbers. However, Twitter exposure had a negative association with both perceived benefits to the household (β=–.058; *F*_1,7589_=24.70; *P*<.001) and community (β=–.030; *F*_1,7592_=5.73; *P*=.02). Weekly exposure to the selected national newspapers is negatively associated with perceived benefits to the household (β=–.085; *F*_1,7592_=44.46; *P*<.001) and to the community (β=–.090; *F*_1,7592_=45.67; *P*<.001). These results suggest that increased exposure to Twitter and the newspapers negatively correlates with perceived benefits of handwashing for both the household and community, rejecting H2 in the handwashing scenario.

Regarding mask-wearing behavior, we found that respondents’ behavioral intention is significantly predicted by resources (β=.196; *F*_1,5288_=369.02; *P*<.001), benefit to the household (β=.164; *F*_1,5064_=149.13; *P*<.001), and benefit to the community (β=.101; *F*_1,5465_=57.09; *P*<.001), supporting H1. Twitter exposure positively influenced perceived household benefits (β=.057; *F*_1,6403_=24.70; *P*<.001) and community benefits (β=.049; *F*_1,6312_=17.35; *P*<.001), while news exposure to the three selected newspapers was positively related to household benefits (β=.031; *F*_1,6442_=6.68; *P*=.009) but negatively to community benefits (β=–.029; *F*_1,6259_=5.34; *P*=.02). These findings were partly in line with H2, which suggests a positive link between media exposure and perceived benefits of mask-wearing behaviors.

For avoiding public gatherings and travel, both perceived benefits for community (β=.044; *F*_1,5698_=5.13; *P*=.02) and for household (β=.348; *F*_1,5723_=280.26; *P*<.001) significantly predicted respondents’ self-reported behavioral intention, supporting H1. Access to resources (β=.157; *F*_1,5688_=171.46; *P*<.001) and increasing weekly case trends (β=.035; *F*_1,4468_=12.78; *P*<.001) also contributed to this avoidance behavior. Twitter (β=.058; *F*_1,5859_=28.62; *P*<.001) and exposure to the three selected national newspapers (β=.031; *F*_1,6458_=6.70; *P*=.009) were positively related to perceived benefits for households, while Twitter exposure alone (β=.051; *F*_1,5979_=20.60; *P*<.001) enhanced perceived community benefits, partially in line with H2.

Resources (β=.338; *F*_1,5559_=757.64; *P*<.001) and perceived household benefits (β=.268; *F*_1,5559_=246.06; *P*<.001) were key predictors of quarantine behavior, which aligns with H1. Perceived community benefits (β=.029; *F*_1,5559_=3.29; *P*=.07) showed a positive but not significant trend. Exposure to the selected national newspapers negatively impacted perceived benefits for both the household (β=–.081; *F*_1,5617_=16.16; *P*<.001) and community (β=–.090; *F*_1,5617_=12.80; *P*<.001), which did not support H2 for quarantine behavior.

Resources (β=.176; *F*_1,5808_=136.69; *P*<.001) and perceived household benefits (β=.377; *F*_1,5808_=280.91; *P*<.001) significantly predicted intentions to vaccinate, suggesting alignment with H1 for household perception. Weekly case trends (β=.026; *F*_1,5808_=5.40; *P*=.02) also positively predicted vaccination intentions. Twitter exposure positively affected the perceived benefits for households (β=.073; *F*_1,7590_=34.03; *P*<.001) and communities (β=.058; *F*_1,7591_=18.36; *P*<.001), indicating support for H2 with respect to social media influence. In contrast, exposure to the selected newspaper sources negatively correlated with household benefits (β=–.026; *F*_1,7590_=4.24; *P*=.04) and was not significant for community benefits (β=–.013; *F*_1,7591_=0.96; *P*=.33), which presents a mixed outcome for H2 when considering different media sources.

Interestingly, an inspection of the random effects analysis highlights that time-related variance in vaccination behavior (σ²=0.016) was lower than individual differences (σ²=0.437). This indicates that differences in vaccination behavior across different time points were relatively small compared to the differences between individuals. The random effects showed substantial variability in baseline vaccination behavior across individuals (σ^2^=0.718), suggesting that some individuals were consistently more inclined to vaccinate than others. Additionally, there was a tendency for those with higher initial intentions to experience smaller changes over time (σ^2^=0.046, corr.=–0.90), implying that people with strong initial intentions to vaccinate were less likely to significantly increase or decrease their intentions across the study period.

## Discussion

### Principal Findings

Our findings advance the literature on health communication in several ways. First, our research contributes to the intermedia agenda-setting theory within the field of health communication that social media platforms, such as Twitter, can set the agenda for some legacy media such as the selected newspaper outlets in our paper, by revealing nuances in patterns across protective behaviors in a pandemic. We noticed no significant agenda flow between the selected newspapers and Twitter in handwashing and mask-wearing, and in avoiding public gatherings. By contrast, there is also a two-way agenda flow—from selected newspapers to Twitter and from Twitter to those newspapers—about vaccination. Contrary to our expectations, the agenda for self-quarantine originated from Twitter and predicted legacy media outlets’ content. That is, our results implied that Twitter users’ discussion on quarantining causes the coverage of quarantine in these legacy media outlets we chose as examples. One explanation could be the massive mental health concerns reflected on social media platforms early in the pandemic in the United States, considering the widespread policy of quarantine at that time [53-55]. These studies identified Twitter (or other social media) users’ mental health concerns at home at an early stage of the pandemic (ie, at or before March 2020). However, it was not until mid-June to July 2020 that there was a boost in these three US top national newspapers’ coverage of mental health issues [56].

Second, our research contributes to the PADM by demonstrating that the relationship between media exposure and protective behavior adoption is contingent upon the specific behavior in question. For certain protective behaviors like avoiding public gatherings and travel and taking up vaccination once vaccines become available, increased exposure to the selected top national newspaper content positively influences individuals’ perceived benefits and behavioral intentions. This aligns with existing literature on the positive impact of media on health behavior [5]. The controversy about vaccinations—such as the misleading vaccine-autism relationship and vaccination mandate debate—mostly happened on social media platforms such as Twitter [18]. Legacy media outlets, on the other hand, have to shoulder the responsibility for communicating scientific and other authorities’ views about appropriate behavior in the pandemic. Therefore, the positive relationship found between exposure to vaccination-related media content and perceived benefit is understandable.

We also see unexpected, but interesting, negative relationships in quarantine and washing hands. People’s perception of the benefits of quarantining might decrease or even backfire in the later stages of the pandemic. This could be due to two factors: (1) mental health concerns arising from social isolation, as previously discussed (eg, [53]), and (2) improvements in nationwide pandemic control [57], leading some states to cancel stay-at-home mandates. Whatever the causes, we observed that respondents’ self-reported practices of quarantine decreased over time. We advance PADM testing by showing that, while exposure to information sources such as national newspapers and Twitter can indeed affect perceptions about protective behaviors and behavioral intentions as the model posits, we find that this impact varies across both behaviors and media platforms. For some behaviors, such as handwashing and avoiding gatherings, both types of media exposure had the same effect on intentions, whereas for others, such as mask-wearing and vaccination, the media platforms did not contribute equally to the public’s behavioral outcomes, with opposite signs for national newspapers and Twitter. There were also differences within a given media type: for instance, while Twitter exposure positively predicts the perceived benefits of mask-wearing and quarantine behaviors, it is negatively associated with the perceived benefits of handwashing in preventing COVID-19. This underscores the need for targeted message strategies that consider platform-specific outcomes to promote public health actions more effectively.

Third, we examined the relationship between media-influenced benefit perception and individuals’ behavioral intention. The positive relationship between perceived benefit and behavioral adoption is within our expectations and is consistent with suggestions from multiple health behavior-related theories, such as the Theory of Planned Behavior [58]. We also noticed negative relationships between the perceived benefits of vaccination and handwashing for the community, and behavioral intentions. Currently, we cannot assertively attribute this phenomenon to a definite theory, but it may be related to egoism versus altruism or prosocial intentions. When individual interests conflict with collective interests in considering vaccination, this situation can be seen as a social dilemma and may result in vaccine hesitation. Our results implied that beliefs about the altruistic effects of these two behaviors undermined intentions, while communication findings elsewhere yielded mixed findings. Altruism-centered messages, aiming to increase individuals’ perceived benefit of vaccination for the community, showed no significant results in increasing vaccination behavior as well [59]. However, while messages conveying self-protection had no effect on vaccine intentions, altruistic messages emphasizing protecting other individuals, population health, and the economy had substantially stronger effects [60], while individuals are responsive to altruistic messaging about vaccination [61].

### Limitations and Future Research

Although we tried to simulate an authentic environment for individuals reading news articles and making decisions, our model cannot replicate the entire decision-making process. The current model suggests that individuals read news articles about protective behaviors, assess their usefulness, consider available resources, check pandemic updates, and then decide whether to adopt these behaviors. However, there are several factors in the PADM missing in this story: for example, it ignores the effects of risk perceptions, or of trust in authorities or the information source, which are also associated with behavioral intentions [36]. There are also limitations in how we calculate the exposure to relevant Twitter content due to the inherent differences in how content is consumed on the platform compared to the national newspaper we selected. In newspaper outlets like the NYT, the number of studies on a given topic is relatively limited and stable, allowing for a straightforward calculation of exposure based on study frequency and reader habits. Twitter’s dynamic, high-volume nature, however, makes a simple multiplication-based metric less meaningful compared to those newspapers.

It is worth noting that the PADM theorists mentioned the factors “should form causal chains” [33], while our statistical test for this model can only convince us of correlation instead of solid causal relationships. The varied patterns in media exposure’s relationship with individuals’ risk perceptions prompt us to reconsider how we filter media content related to protective behaviors. We focused only on the content discussed, specifically whether keywords were mentioned (first step of dictionary-based filtering) and whether the topic was health-related (second step of topic-based filtering). We did not look into how they discussed these protective behaviors. For example, media coverage about vaccination could vary in tone as the development of effective COVID-19 vaccines evolved from uncertain to clinically proven during our six survey waves [62].

Additionally, this study is US-based, with a US national survey and US national newspapers in analyses, which limits the generalizability of our findings to a global perspective. Although we collected tweets in English and aimed to capture US users’ conversations, our dataset may include tweets from English-speaking users outside the United States due to limitations in geo-tagging availability. This introduces a degree of uncertainty in assuming that all social media data reflect US perspectives. Furthermore, there are well-documented differences in public risk perceptions and protective behaviors across countries, influenced by sociocultural factors such as individualistic or prosocial values, trust in authorities, and personal experience with the virus [63]. When applying the US-based findings to other contexts, we cannot ignore the role of cross-cultural variations and how they differently shape people’s responses to health risks.

Communication during the COVID-19 pandemic has faced a concern of an “infodemic” with wicked challenges like misinformation, resistance, and fear [64,65]. Exposure to this infodemic might further elicit individuals’ worries about uncertainty and information-seeking behavior [66,67]. Therefore, we believe studying the agenda consistency between different media platforms is essential for effective pandemic management. However, scholars have long been concerned that media can have multiple roles in communicating risk-protective behaviors to lay audiences, and different channels (eg, legacy media vs nonlegacy media such as social media) can have different communication effects [4]. The transmission of quarantine agendas from Twitter to the selected top national newspapers, as observed in this study for two of the five behaviors, along with the differing perceived benefits for household versus community protective behaviors, caution practitioners to consider multiple factors that may be salient to audiences when promoting protective behaviors. This study also calls for further steps for building up more profound and solid models in predicting individuals’ behavioral intentions in health communication research.

Our findings carry several actionable strategies for health communicators and decision makers to organize and publish media content. First, practitioners can adopt a platform-specific approach. Social media platforms like Twitter and legacy media such as the national newspapers can influence public perceptions in different ways. For Twitter, practitioners should stay aware of trending conversations about key health behaviors (eg, vaccinating) and engage in addressing misinformation or reinforcing protective behaviors, including changes over time (eg, [68]). Such engagement can help in spreading accurate information and serve as a feedback mechanism with metrics such as engagement rates.

Second, although exposure to some legacy media outlets (eg, national newspapers) can raise awareness and perceived benefits of protective behaviors like vaccination, our data show that this awareness does not always translate into self-reported behavior change. For example, high newspaper exposure was associated with lower perceived household and community benefits for quarantine, even though these perceived benefits were a positive indicator of behavioral change. Therefore, practitioners should design campaigns that not only raise awareness but also emphasize actionable steps of specific protective behaviors, such as tailoring clear instructions and feasible solutions to barriers. Public opinion shows there are barriers for individuals to fully follow “safer at home” policies, including emotional strain and practical challenges [69]. By acknowledging these issues, communicators can offer practical solutions and support, enhancing effectiveness, other than merely increasing the behavior’s presence in media.

Third, increased media exposure can sometimes have unintended effects by reinforcing negative perceptions, as seen in our findings where both Twitter and newspaper exposure were linked to lower perceived benefits of handwashing. To avoid these unintended consequences, practitioners should pretest messages with diverse audiences to identify potential misinterpretations or negative framing before widespread dissemination. Additionally, focusing on clear, fact-based messaging that emphasizes practical benefits and personal relevance can help reduce skepticism. The overwhelming amount of unclear, ambiguous, and inaccurate information during the early stage of the COVID-19 pandemic contributed to information overload, which heightened health-related anxiety and increased the spread of misinformation [70,71]. Clear communication can help counteract these negative effects by providing trustworthy, straightforward guidance.

Finally, it is important to consider whether the heightened media consumption during the global pandemic translates into healthy communication strategies for more “normal” times, when media use may be more fragmented. Most of our survey respondents (85%) reported in the first wave that they were using their usual media channels for COVID-19 information, with the exceptions primarily COVID-19–specific outlets (eg, US CDC and World Health Organization websites). Furthermore, unpublished analyses of patterns of self-reported media exposure across different media types (eg, the most common was moderate use of national newspapers, digital news, and social media) found that about 10% of people shifted these patterns from one wave to another, again suggesting that there was mainly stability in media use from before to during the pandemic through April 2021, the last point at which we collected these data. However, media consumption patterns may shift post pandemic, requiring communicators to adjust their strategies.

### Conclusions

While news consumption surged during the pandemic [72], postpandemic media engagement has stabilized but remains above prepandemic levels, and audiences are engaging with more new technology [73,74]. Considering this fragmented attention, health communication practitioners should focus on targeting specific platforms, moving away from blanket exposure and toward tailored content that resonates with diverse, targeted audiences [75]. Practitioners should adapt by segmenting audiences and leveraging analytics to track and optimize message delivery across different platforms.

## Appendix

Multimedia Appendix 1 Keywords for protective behaviors.

Multimedia Appendix 2 Topic modeling process for filtering irrelevant news articles and tweets.

Multimedia Appendix 3 Key survey constructs used for measuring media exposure, perceived benefit, recourses, and behavioral intention.

Multimedia Appendix 4 How media exposure was calculated.

Multimedia Appendix 5 Updated table for the regression.

## Abbreviations

CDC: Centers for Disease Control and Prevention
NYT: New York Times
PADM: Protective Action Decision Model
WSJ: Wall Street Journal

## Data Availability

The dataset analyzed in this study is publicly available in the Interuniversity Consortium for Political and Social Research repository [76].
