# Supplementary material for: Ubiquitous News Coverage and Its Varied Effects in Communicating Protective Behaviors to American Adults in Infectious Disease Outbreaks: Time-Series and Longitudinal Panel Study
Source: J Med Internet Res. 2025 Mar 10;27:e64307. doi: 10.2196/64307 (PMC11933775; doi:10.2196/64307)
Supplement: Multimedia Appendix 1 [file jmir_v27i1e64307_app1.docx]

| Category | Search String |
| --- | --- |
| Wash hands | (wash hand*)\|(hand wash*)\|(hand sani*) |
| Wear masks | (mask)\|(face cover*)\|(mask wear)\|(facemask) |
| Self or mandatory Quarantine | (quarantin*)\|(stay at home)\|(stay-at-home)\|(isolat*)\|(social distanc*)\|(social-distanc*) |
| Avoid gathering and travel | (avoid travel)\|(avoid public)\|(avoid crowd*)\|(avoid gather*)\|(avoid people)\|(avoid others)\|(social distanc*)\|(social-distanc*) |
| Take vaccination | (vaccin*) |
