# Supplementary material for: Ubiquitous News Coverage and Its Varied Effects in Communicating Protective Behaviors to American Adults in Infectious Disease Outbreaks: Time-Series and Longitudinal Panel Study
Source: J Med Internet Res. 2025 Mar 10;27:e64307. doi: 10.2196/64307 (PMC11933775; doi:10.2196/64307)
Supplement: Multimedia Appendix 2 [file jmir_v27i1e64307_app2.docx]

We used structural topic modeling (STM) to filter irrelevant news articles and tweets related to protective behaviors. The optimal number of topics for the news articles was determined to be K=47, based on a combination of metrics: among the highest held-out likelihood, the smallest residuals, a high lower bound, and good semantic coherence. For Twitter content, K = 20 topics were identified.

During the data filtering process, three authors manually contributed to topic identification and evaluated the relevance of each topic. For the news articles, 22 of the 47 topics were deemed relevant to protective behaviors, while 12 of the 20 topics for Twitter content were considered relevant. Irrelevant topics were excluded from further analysis of media coverage and exposure.

To validate the relevance of the filtered content that were considered relevant with keyword and topic models, we conducted a manual review of a representative sample. For news articles, we randomly reviewed 211 articles (2% of the filtered sample) and found that 96.68% were closely related to health topics. For tweets, we examined 209 randomly selected tweets (0.1% of the filtered sample) and found 95.69% were directly relevant to protective behaviors. The small percentage of irrelevant content included discussions on unrelated topics, such as geopolitical events, immigration, and politics.

Below are two examples of news article snippets that we included and excluded with topic modeling and their FREX (frequent and exclusive) keywords, which helped identify the topics most relevant to protective behaviors.

**Included example**

*“New York City is recording a slow and steady rise in Covid-19 cases, Mayor Bill de Blasio said Thursday, with health officials blaming some of the uptick on out-of-state travelers and indoor gatherings.*

*…*

*Dr. Varma said the decision to impose lockdown restrictions on the hot spots earlier this month helped drive down cases. While the rise has been relatively slow since then, he said, city officials are concerned that it hasn't fully abated.*

*""Right now our guidance is not about one specific industry or type of setting changing its behavior, but it really has to be across the city,"" Dr. Varma said. ""Wearing a mask, avoiding any gatherings, keeping physical distance, particularly as it gets colder and people move indoors.""*

*The mayor on Thursday repeated what he said earlier this week, advising New Yorkers to avoid traveling during the holidays unless it is absolutely necessary. On Tuesday, he said the city will aggressively enforce a 14-day quarantine for people traveling from 39 states and the two U.S. territories of Guam and Puerto Rico, where coronavirus cases are rising at higher rates.*

*Mr. de Blasio warned that city officials would break up large gatherings or indoor events during Halloween this weekend, but said outdoor trick-or-treating in small groups while wearing masks is safe.”*

**Topic FREX keywords:** death, case, toll, surg, averag, peak, hopkin, number, daili, seven-day

**Decision:** This topic is about pandemic outlook within U.S. It is relevant to the protective behaviors, and we should include it.

**Excluded example**

*Revlon Inc. reported a decline in first-quarter sales amid the pandemic's continuing drag on demand for makeup.*

*The New York-based cosmetics company posted first-quarter sales of $445 million, down from $453 million in the year-ago quarter.*

*The pandemic cut revenue by about $44 million, Revlon estimated. Sales of cosmetics have been lower since last year as lockdowns and social distancing reduced occasions such as business meetings and parties.*

*For the quarter, the company posted a total loss of $97.4 million, compared with a loss of $216.6 million in the year-earlier quarter.*

*A restructuring program has helped reduce Revlon's costs, the company said, as selling, general and administrative expenses declined by $28.9 million year over year. The program aims to focus on boosting the company's Revlon and Elizabeth Arden brands in key markets and expanding e-commerce capabilities.*

**Topic FREX keywords:** retail, e-commerc, shopper, lyft, sale, walmart, uber, auto, doordash, apparel

**Decision:** This topic is about sales and business. Although it contains “social distancing” keywords, it is largely irrelevant to the protective behaviors, and we should exclude it.
