# Supplementary material for: Ubiquitous News Coverage and Its Varied Effects in Communicating Protective Behaviors to American Adults in Infectious Disease Outbreaks: Time-Series and Longitudinal Panel Study
Source: J Med Internet Res. 2025 Mar 10;27:e64307. doi: 10.2196/64307 (PMC11933775; doi:10.2196/64307)
Supplement: Multimedia Appendix 3 [file jmir_v27i1e64307_app3.docx]

**Media exposure**

How often do you use each of the following media outlets for information specific to the coronavirus? (1-7 scale reversely coded from many times a day, daily, three times a week, once per week, monthly, rarely, never)

|  | many times a day | daily | three times a week | once per week | monthly | rarely | never |
| --- | --- | --- | --- | --- | --- | --- | --- |
| National newspapers and news magazines (online & offline), like The New York Times, USA Today, or the Wall Street Journal |  |  |  |  |  |  |  |
| Social media platforms, like Facebook or Twitter |  |  |  |  |  |  |  |

**Perceived benefit for household and community (e.g., vaccination)**

Action: Getting vaccinated when a coronavirus vaccine becomes available

|  | Strongly disagree | Disagree | Somewhat disagree | Somewhat agree | Agree | Strongly agree |
| --- | --- | --- | --- | --- | --- | --- |
| This action will keep coronavirus risks low for my household. |  |  |  |  |  |  |
| This action will keep coronavirus risks low for vulnerable people in my community not in my household. |  |  |  |  |  |  |

**Perceived resources for action (e.g., vaccination)**

Action: Getting vaccinated when a coronavirus vaccine becomes available

|  | Strongly disagree | Disagree | Somewhat disagree | Somewhat agree | Agree | Strongly agree |
| --- | --- | --- | --- | --- | --- | --- |
| My household has the time, money, skills and/or other resources needed to take this action. |  |  |  |  |  |  |

**Behavioral outcomes (e.g., vaccination)**

Action: Getting vaccinated when a coronavirus vaccine becomes available

My household...

- has never considered taking this action
- is considering it
- decided against taking this action
- decided to take this action
- has taken this action
- has taken this action and will continue to take this action as needed
