# Supplementary material for: Ubiquitous News Coverage and Its Varied Effects in Communicating Protective Behaviors to American Adults in Infectious Disease Outbreaks: Time-Series and Longitudinal Panel Study
Source: J Med Internet Res. 2025 Mar 10;27:e64307. doi: 10.2196/64307 (PMC11933775; doi:10.2196/64307)
Supplement: Multimedia Appendix 4 [file jmir_v27i1e64307_app4.docx]

Table S1 below provides an intuitive explanation of how individuals’ media content exposure for selected national newspapers was calculated for a one-week time window. Media exposure for Twitter was calculated with the same method.

**Table S1.** Example procedure of how we calculated the media exposure for each respondent in each wave of survey with a one-week time window.

|  | 1 week time window before the survey response date | | | | | | |  |
| --- | --- | --- | --- | --- | --- | --- | --- | --- |
|  |  | | | | | | | Response date |
| Date | Day -7 | Day -6 | Day -5 | Day -4 | Day -3 | Day -2 | Day -1 | Day 0 |
| Daily volume of total newspaper articles | $N_{7}$ | $N_{6}$ | $N_{5}$ | $N_{4}$ | $N_{3}$ | $N_{2}$ | $N_{1}$ |  |
| Daily volume of articles on this topic | $n_{7}$ | $n_{6}$ | $n_{5}$ | $n_{4}$ | $n_{3}$ | $n_{2}$ | $n_{1}$ |  |
| Respondent’s exposure frequency to national newspapers in this wave | $f$ | | | | | | |  |
| Respondents’ weekly media content exposure | $\frac{\sum_{i=1}^{7} n_{i}}{\sum_{i=1}^{7} N_{i}}$ $\cdot f$ | | | | | | |  |

Assume a participant responded to the Wave 2 survey on April 28, 2020, as an example. The date range for calculating their weekly media exposure would be April 21 – April 27, 2020, representing the full week before they filled in this response. For each day this week, assume that a participant randomly browses through^[[1]](#footnote-1)^ all the media contents in national newspapers. The likelihood that they are exposed to topic-relevant contents (e.g., if the topic is vaccination) is calculated as the proportion of articles on vaccination relative to the total daily volume of newspaper articles. This likelihood is then summed across the week and multiplied by their self-reported exposure frequency (with higher values indicating more frequent exposure). This results in the metric for their weekly media content exposure used in this study.

1. We acknowledge the limitation here as in reality, people’s attention to news content is not evenly distributed. For example, they may use media for specific purposes such as look up for updates about vaccines or mask mandates, instead of randomly browsing all contents published out there. [↑](#footnote-ref-1)
