# Supplementary material for: Ubiquitous News Coverage and Its Varied Effects in Communicating Protective Behaviors to American Adults in Infectious Disease Outbreaks: Time-Series and Longitudinal Panel Study
Source: J Med Internet Res. 2025 Mar 10;27:e64307. doi: 10.2196/64307 (PMC11933775; doi:10.2196/64307)
Supplement: Multimedia Appendix 5 [file jmir_v27i1e64307_app5.docx]

**Table S1:** Estimated coefficients from mixed-effect regression models of respondents’ behavioral association with media coverage, perceived benefit, and other controlling variables. For each protective behavior, BIntn refers to behavioral intention, PHBf refers to perceived household benefit, and PCBf refers to perceived community benefit.

|  | | | | | | | | | | | | | | | |
| --- | --- | --- | --- | --- | --- | --- | --- | --- | --- | --- | --- | --- | --- | --- | --- |
|  | *Protective behaviors* | | | | | | | | | | | | | | |
|  |  | | | | | | | | | | | | | | |
|  | Wash hands | | | Wearing masks | | | Avoid public gatherings | | | Isolation/quarantine | | | Get vaccinated | | |
|  |  |  |  |  |  |  |  |  |  |  |  |  |  |  |  |
|  |  |  |  |  |  |  |  |  |  |  |  |  |  |  |  |
|  | BIntn | PHBf | PCBf | BIntn | PHBf | PCBf | BIntn | PHBf | PCBf | BIntn | PHBf | PCBf | BIntn | PHBf | PCBf |
|  | | | | | | | | | | | | | | | |
| Resources | 0.098^***^ |  |  | 0.196^***^ |  |  | 0.157^***^ |  |  | 0.338^***^ |  |  | 0.176^***^ |  |  |
|  | (0.012) |  |  | (0.010) |  |  | (0.012) |  |  | (0.012) |  |  | (0.015) |  |  |
|  |  |  |  |  |  |  |  |  |  |  |  |  |  |  |  |
| Perceived household benefit | 0.294^***^ |  |  | 0.164^***^ |  |  | 0.338^***^ |  |  | 0.268^***^ |  |  | 0.377^***^ |  |  |
|  | (0.016) |  |  | (0.013) |  |  | (0.020) |  |  | (0.017) |  |  | (0.023) |  |  |
|  |  |  |  |  |  |  |  |  |  |  |  |  |  |  |  |
| Perceived community benefit | -0.010 |  |  | 0.101^***^ |  |  | 0.044^**^ |  |  | 0.029^*^ |  |  | -0.048^**^ |  |  |
|  | (0.015) |  |  | (0.013) |  |  | (0.019) |  |  | (0.016) |  |  | (0.021) |  |  |
|  |  |  |  |  |  |  |  |  |  |  |  |  |  |  |  |
| Weekly trend of case update | 0.024^**^ |  |  | 0.050^***^ |  |  | 0.035^***^ |  |  | -0.010 |  |  | 0.026^**^ |  |  |
|  | (0.010) |  |  | (0.007) |  |  | (0.010) |  |  | (0.011) |  |  | (0.011) |  |  |
|  |  |  |  |  |  |  |  |  |  |  |  |  |  |  |  |
| Weekly exposure to Twitter contents |  | -0.058^***^ | -0.030^**^ |  | 0.057^***^ | 0.049^***^ |  | 0.058^***^ | 0.051^***^ |  | 0.020 | 0.013 |  | 0.057^***^ | 0.049^***^ |
|  |  | (0.012) | (0.013) |  | (0.011) | (0.012) |  | (0.011) | (0.011) |  | (0.014) | (0.015) |  | (0.011) | (0.012) |
|  |  |  |  |  |  |  |  |  |  |  |  |  |  |  |  |
| Weekly exposure to selected newspapers |  | -0.085^***^ | -0.090^***^ |  | -0.023^*^ | -0.029^**^ |  | 0.031^***^ | 0.023^*^ |  | -0.081^***^ | -0.077^***^ |  | -0.023^*^ | -0.029^**^ |
|  |  | (0.013) | (0.013) |  | (0.012) | (0.013) |  | (0.012) | (0.012) |  | (0.020) | (0.021) |  | (0.012) | (0.013) |
|  |  |  |  |  |  |  |  |  |  |  |  |  |  |  |  |
| Constant | -0.039 | -0.019 | -0.107 | 0.368^***^ | 0.037 | -0.048 | 0.028 | 0.267^***^ | 0.185^**^ | 0.004 | 0.039 | -0.097 | -0.047 | 0.037 | -0.048 |
|  | (0.076) | (0.085) | (0.082) | (0.038) | (0.075) | (0.073) | (0.065) | (0.086) | (0.084) | (0.072) | (0.092) | (0.090) | (0.068) | (0.075) | (0.073) |
|  |  |  |  |  |  |  |  |  |  |  |  |  |  |  |  |
|  | | | | | | | | | | | | | | | |
| Observations | 5,812 | 7,592 | 7,592 | 5,809 | 7,589 | 7,589 | 5,814 | 7,594 | 7,595 | 5,559 | 5,617 | 5,617 | 5,808 | 7,589 | 7,589 |
| Log Likelihood | -6,369.610 | -9,639.871 | -10,072.650 | -4,349.030 | -9,976.829 | -10,191.470 | -6,067.595 | -9,801.482 | -9,974.874 | -6,218.553 | -6,847.312 | -7,181.282 | -6,989.958 | -9,976.829 | -10,191.470 |
| Akaike Inf. Crit. | 12,779.220 | 19,315.740 | 20,181.290 | 8,738.059 | 19,989.660 | 20,418.950 | 12,175.190 | 19,638.960 | 19,985.750 | 12,477.110 | 13,730.620 | 14,398.570 | 14,019.920 | 19,989.660 | 20,418.950 |
| Bayesian Inf. Crit. | 12,912.570 | 19,440.570 | 20,306.120 | 8,871.403 | 20,114.480 | 20,543.770 | 12,308.550 | 19,763.790 | 20,110.580 | 12,609.570 | 13,850.030 | 14,517.970 | 14,153.250 | 20,114.480 | 20,543.770 |
|  | | | | | | | | | | | | | | | |
| *Note:* | ^*^p<0.1; ^**^p<0.05; ^***^p<0.01 | | | | | | | | | | | | | | |
